# Assessment of Simple Models for Molecular Simulation of Ethylene Carbonate and Propylene Carbonate as Solvents for Electrolyte Solutions

**DOI:** 10.1007/s41061-018-0187-2

**Published:** 2018-02-12

**Authors:** Mangesh I. Chaudhari, Ajay Muralidharan, Lawrence R. Pratt, Susan B. Rempe

**Affiliations:** 1 Center for Biological and Engineering Sciences, Sandia National Laboratories, Albuquerque, NM 87185 USA; 2 Department of Chemical and Biomolecular Engineering, Tulane University, New Orleans, LA 70118 USA

**Keywords:** Li-ion battery, Molecular dynamics simulations, Propylene carbonate, Ethylene carbonate

## Abstract

Progress in understanding liquid ethylene carbonate (EC) and propylene carbonate (PC) on the basis of molecular simulation, emphasizing simple models of interatomic forces, is reviewed. Results on the bulk liquids are examined from the perspective of anticipated applications to materials for electrical energy storage devices. Preliminary results on electrochemical double-layer capacitors based on carbon nanotube forests and on model solid-electrolyte interphase (SEI) layers of lithium ion batteries are considered as examples. The basic results discussed suggest that an empirically parameterized, non-polarizable force field can reproduce experimental structural, thermodynamic, and dielectric properties of EC and PC liquids with acceptable accuracy. More sophisticated force fields might include molecular polarizability and Buckingham-model description of inter-atomic overlap repulsions as extensions to Lennard-Jones models of van der Waals interactions. Simple approaches should be similarly successful also for applications to organic molecular ions in EC/PC solutions, but the important case of Li$^+$ deserves special attention because of the particularly strong interactions of that small ion with neighboring solvent molecules. To treat the Li$^+$ ions in liquid EC/PC solutions, we identify interaction models defined by empirically scaled partial charges for ion-solvent interactions. The empirical adjustments use more basic inputs, electronic structure calculations and ab initio molecular dynamics simulations, and also experimental results on Li$^+$ thermodynamics and transport in EC/PC solutions. Application of such models to the mechanism of Li$^+$ transport in glassy SEI models emphasizes the advantage of long time-scale molecular dynamics studies of these non-equilibrium materials.

## Introduction

An electrochemical voltage window is a primary concern for electrical energy storage applications of an electrolyte system, e.g., for lithium ion batteries (LIBs) and electrochemical double-layer capacitors (EDLCs). That voltage window is a primary issue for the energy density, but also a consideration in addressing safety. As a practical matter, that voltage window concern excludes aqueous electrolyte solutions [1]. Non-aqueous electrolyte solutions [2] are well-recognized, but the molecular simulation experience with those systems is orders of magnitude more limited than for aqueous systems [3–5]. This is partly due to the broad importance of water as a liquid medium [6], but also due to vast chemical and compositional variety relevant for non-aqueous systems [7–10].

Understanding that daunting range of chemical possibilities, including assessment of voltage windows, has put natural emphasis on screening enabled by electronic structure computations of theoretical chemistry [11–13]. But macroscopic characteristics of these liquids—such as phase diagrams, dielectric responses, and fluid phase kinetics—are relevant too, and direct numerical simulation of the solutions help in that screening. Careful molecular simulation often requires validation of models and techniques, consideration of a range of thermodynamic states, and understanding the scale limitations of the results. Therefore it can be helpful for simulation work to examine relevant cases in depth to complement screening approaches.

**Fig. 1:**
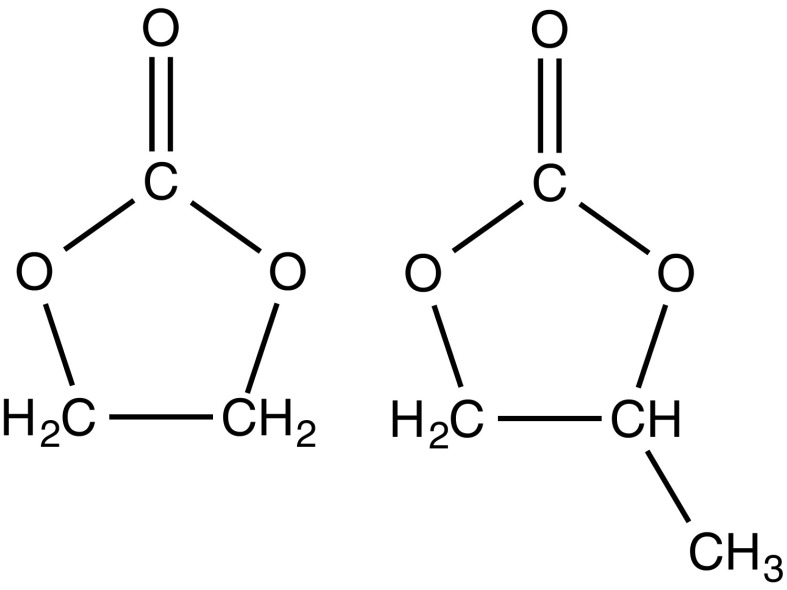
Chemical structures of ethylene carbonate (EC) and propylene carbonate (PC)

Recent work has aimed at filling in the simulation basis for study of non-aqueous electrolyte solutions, i.e., for ethylene carbonate (EC) and propylene carbonate (PC) systems, at a molecular level (Fig. 1). This report collects and discusses recent simulation results on these solvents to identify basic research that might help in further design of materials.

Molecular simulation is a useful tool for development of new materials. Development of effective, simplified molecular simulation models would enable enlightening simulation of dynamical phenomena of specific interest for electrical energy storage systems, i.e., transport through inhomogeneous or non-equilibrium materials, or of annealing processes involving those non-equilibrium materials.

Electrochemical double-layer capacitors (EDLCs), or supercapacitors [14], present cases of inhomogeneous materials. EDLCs based on carbon nanotube (CNT) forests provide a specific setting for molecular-scale examination of the dynamics of propylene carbonate solutions of complex salts [15], a setting where the underlying microstructure is comparatively unambiguous [16]. We note that a variety of solvent/electrolyte systems, including ionic liquids [10], are commonly considered with EDLCs. Nevertheless, molecular-scale descriptions that might explain the dependence on electrode microscale structures or of the rates of charging/discharging have not been carried through. Synthesis of CNTs [17–19] with well-characterized molecular-scale microstructure should assist in establishing the molecular theories sought to understand these systems fully.

An even more prominent example of important molecular-scale kinetics involving inhomogeneous or non-equilibrium materials is that of Li$^+$ ion transport through the solid electrolyte interphase (SEI) of lithium ion battery (LIB) [20]. The SEI of an LIB forms during initial charging/discharging cycles [21–23]. Solution components decompose [24], forming a passivating anode layer. Li$^+$ ions travel through that complex organic material. The composition of the SEI depends on a variety of factors, including solvent and additives, ions, anode material, voltage, temperature and the use history. Understanding the atomic-scale mechanism of transport of Li$^+$ ion through the SEI should assist in development of high-performance LIBs, through better characterization and control of the SEI layer. Molecular simulations might help to bridge the learning gap [8, 9, 25, 26].

Molecular calculations and simulations are typically a necessary prerequisite for basic molecular theories. Molecular calculations span a daunting range of algorithmic techniques, and a daunting range of space and time scales. For example, quantum calculations track electrons and can characterize decomposition of electrolytes at anode surfaces [8, 9, 22, 27, 28]. These methods include ab initio molecular dynamics (AIMD), which have the drawback of computational expense and the concomitant limitation to small systems and time scales [29–31]. On the other hand, if chemical changes such as chemical bond rearrangement are essential to the study, AIMD provides natural perspectives on those phenomena.

Classical molecular dynamics simulation with model molecular force fields—‘force field molecular dynamics’ (FFMD)—inhabit a broader region of the simulation scale. Useful force field models can span a broad range of possibilities, from frankly ad hoc models, to models that are recognized as coarse-grained on a pragmatic basis, then including progressively more complicated models. Electron coordinates can be reintroduced into FFMD approaches by development of models that include molecular polarizabilities. Polarization of that type has been considered important for this problem [32]. The polarizable force field of Borodin et al. [7]. for the carbonate solvents and SEI layer models [25, 26], has been applied to LiBF$_4$ in PC [34].

Highly specialized force fields and parameters are not readily available for common molecular simulation packages. Additionally, their complexity limits their use for study of transport behaviors for novel materials that interrogate molecularly long correlation times. This overview focuses on the pragmatic middle of FFMD simulations focused on non-polarizable force fields with empirical parameters.

### Methods and Force Fields

The FFMD simulations discussed specifically here were carried out using the GROMACS simulation package [35]. Details of the calculations differ slightly between cases, as noted with those discussions below, but were always obtained with force fields of standard non-polarizable format. We note the success of empirical force fields for liquid water [3–5]. EC and PC liquid results here used all-atom optimized potentials for liquid simulation (OPLS-AA) force fields and parameters [36]. There were several distinct reasons for these choices, beginning with simplicity and accessibility of these molecular simulation basics. Another reason for the present simple choice of force field model is that we emphasize liquid phase thermal properties that are statistical challenges for molecular simulation. Thus, the ability to examine sufficiently long statistical series is an important consideration. Finally, we note the sufficiency of empirical potential structure refinement (EPSR) modeling for reproducing the neutron diffraction results with exactly the same forms [37]. Thus, the present simple force field models should be sufficient also for those important data.

We identify secondary specific differences among those FFMD calculations discussed below, but we here provide several common features. These calculations adopted constant pressure simulation conditions with *p* = 1 atm on the basis of the Parrinello-Rahman barostat [38]. Temperatures were maintained with a Nosé-Hoover thermostat [39, 40]. A time step of 1 fs and time constant of 2.5 ps were used for the thermostat and barostat, respectively. Periodic boundary conditions were applied standardly to simulate bulk liquid conditions. The particle mesh Ewald method was used to compute electrostatic interactions, and Lennard-Jones interactions were cut-off at 1.2 nm. Long-ranged dispersion corrections were also applied. Bonds involving hydrogen atoms were constrained using the linear constraint solver (LINCS) algorithm [41].

Extensions of such a simple force field model are interesting for the chemical physics of these problems. A Buckingham [32, 42] model of van der Waals repulsions is an extended feature that is likely to be generally helpful compared to a traditional $1/r^{12}$ (Lennard-Jones) model. We comment further about that extension below when we discuss solvation of Li$^+$ ions in these carbonate liquids. Another interesting extension is the inclusion of solvent molecular polarizability in these force fields [32, 42]. This feature is likely to be specifically important for electrolytes—involving free ions—but we emphasize that dispersive van der Waals interactions are modeled separately in these forces fields. We note in passing that establishment of saturated solution conditions, perhaps involving ion pairing as a mechanism for phase separation, is primarily sensitive to (attractive) dispersive van der Waals interactions [43–45]. As with the common empirical force fields for liquid water simulation [3–5], the non-polarizable force field parameters should be recognized as effective values that approximate the outcomes obtained with more complicated force fields.

### Plan of this Report

We will collect and discuss molecular simulation results on the EC and PC liquids and on solutions with electrolytes relevant to EDLC capacitors based on CNT forests [44, 46–48]. We will emphasize macroscopic characteristics that are often considered in discussions of such applications, particularly interfacial structure, dielectric responses, and molecular mobilities. In focusing here on the molecular basis of macroscopic characteristics of these liquids, the present report aims to complement the recent review [49] that emphasized synthesis and catalysis. We take up the example of EDLCs based on CNT forests where the liquid carbonate solutions are integral components. We then include Li$^+$ in these calculations [32, 50], leading to discussion of simulation of a model LIB SEI layer [42].

In closing this introduction, we reemphasize the common goal of devising high-capacity, fast-charging, safe electrical energy storage systems [21]. Commonly used electrical energy storage devices do present distinct material requirements. Therefore, breadth and fidelity in understanding possible materials should be an advantage. Indeed, other solvents have been considered in this context. For example, glycerol carbonate has been studied recently by neutron diffraction and modeling [37]. Nitriles have received extended study [1, 51–53], as has acetonitrile [54–62]. Of course, it is the non-aqueous conditions that are of interest here. But LIB applications have involved carbonate solvents, instead of nitriles [1], because of the role of carbonate molecules in chemical processes that form the SEI [63]. A recent study of PC/acetonitrile mixtures is striking due to the unusual solvent combination [64].

**Fig. 2:**
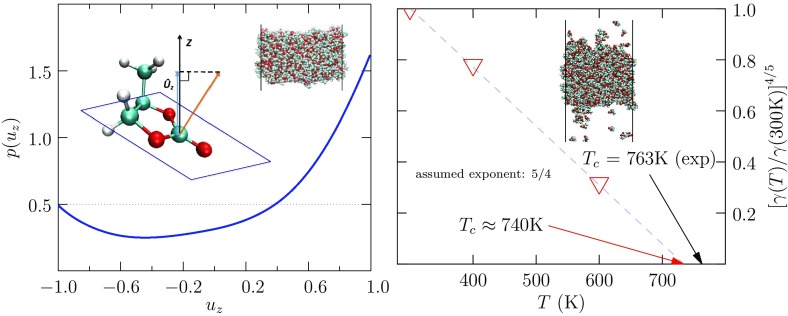
(*left*) For PC molecules in the liquid-vapor interfacial layer at $T=300$ K, the probability density for projection of the unit vector normal to the carbonate plane onto the axis, perpendicular to the interface. The *inset* in upper-right corner of that panel indicates the slab geometry used for these calculations [44, 65]. The ‘outer’ (vapor) direction corresponds to projections near 1.0. The most probable orientation aligns the carbonate plane parallel to the plane of the interface, with the methyl group extended toward the vapor phase. $u_z > 0.5$ ($\theta < 60^\circ$) for about 50% of interfacial PC molecules. (*right*) Liquid-vapor interfacial tensions for PC, extrapolated to estimate the critical temperature $T_\mathrm{c}$ as shown. The surface tension for the lowest *T* shown here agrees well with the one experimental evaluation of that tension at $T=20^\circ$ C. The estimated vapor pressures for these cases are roughly correct [66]. The *inset* on the *right panel* is a configuration drawn from the $T=600$ K calculation, which thus gives an indication of the co-existing vapor. These results together provide support for the observed interface structures and suggest that the balance of attractive intermolecular interactions is realistic

## Ethylene Carbonate and Propylene Carbonate Liquids

The vapor pressures of EC and PC are low in regimes of practical use [44, 66], and thus they are strongly bound liquids. We further characterize [46] this ‘strongly bound’ quality by the ratio $T_\mathrm {c}/T_\mathrm {t}$ of their critical temperatures to their triple-point temperatures. For the well-studied Lennard-Jones model liquid, this ratio is $T_\mathrm {c}/T_\mathrm {t}=1.9$. But for liquid PC and EC, this ratio is 3.5 (PC) and 2.3 (EC). Acetonitrile and water, for which $T_\mathrm {c}/T_\mathrm {t} \approx 2.4$, provide further comparisons [46].

**Fig. 3:**
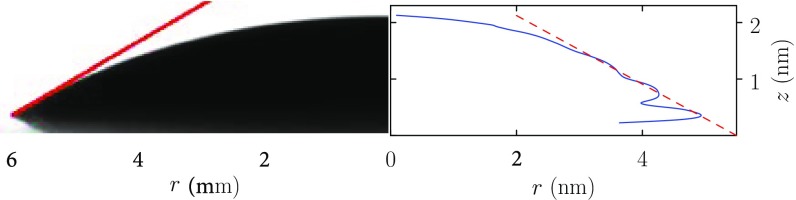
PC droplet on graphite. The *left side* is the observed millimeter-scale droplet [44]. The *blue curve* on the *right side* is the nanometer-scale simulated droplet shape, obtained with adjustment of the van der Waals interaction to match the experimental contact angle as described in that reference. The *fringe* on the *right side* of the simulated droplet illustrates nanometer-scale molecular layering of PC molecules in contact with the graphite surface

**Fig. 4:**
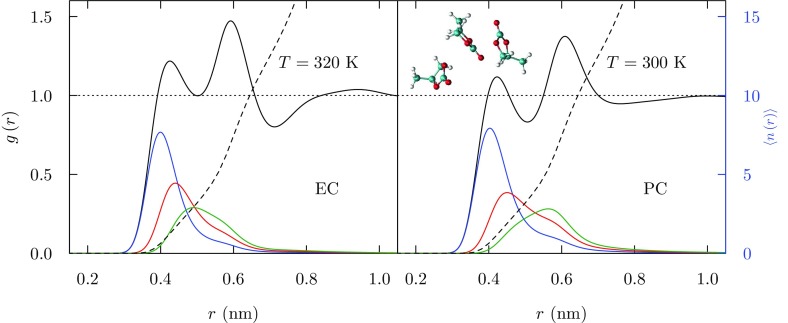
Radial distribution functions associated with carbonyl carbon atoms. (*black*): Traditional radial distributions involving all carbonyl carbon atoms with the characteristic split primary peak, further quantified by the neighborship-ordered radial distribution functions for the closest (*blue*), 2nd-closest neighbors (*red*), and 3rd-closest neighbors (*green*) of a carbonyl carbon atom. The embedded molecular graphic illustrates that these close pairs exhibit stacking of carbonate planes and antiparallel dipole moments. The two closest neighbors saturate the closest peak of the traditional distribution function, and stacking of one plane on top and another on bottom achieves that

Estimation of $T_\mathrm {c}$ for our standard simulation model of PC, on the basis of extrapolation of liquid-vapor surface tensions (Fig. 2), is remarkably accurate. Study of those interfaces shows that the plane of the PC molecular is statistically oriented parallel to the interfacial plane, with the methyl group directed toward the vapor phase. Planar stacking persists when liquid PC contacts a planar graphite surface (Fig. 3), except that the methyl group is preferentially oriented toward the liquid phase in that case [44]. The relative orientation of near-neighbor PC molecules in the liquid (Fig. 4) again display this rough planar stacking motif, with the two nearest neighbors corresponding to a plane above and a plane below a distinguished PC molecule. The dipole moments of these stacked neighbors tend to be anti-parallel and this has by now been experimentally confirmed on the basis of neutron diffraction from PC and glycerol carbonate [37].

The liquid density of standardly simulated PC at $p = 1$ atm is several percent denser [44] than the experimental value near $T=300$ K. The thermal expansion coefficient from the simulations under those conditions is highly accurate, although the isothermal compressibility is too small by about 50% [44]. We expect that discrepancy in the isothermal compressibility would be improved by effective replacement of Lennard-Jones $1/r^{12}$ repulsions by Buckingham repulsions [32, 42].

**Fig. 5:**
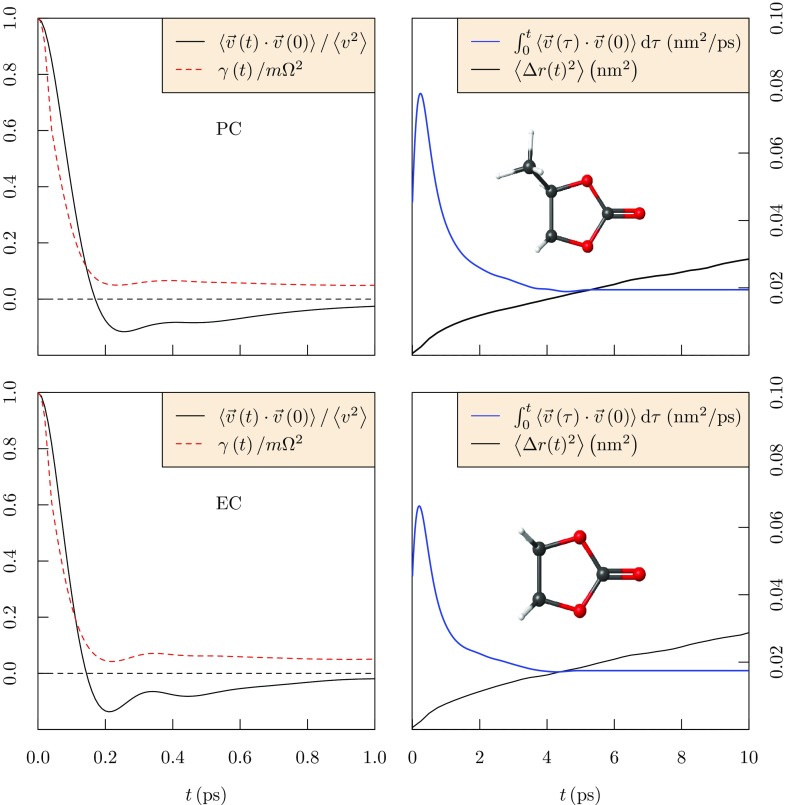
(*top-left*) Time correlation functions associated with the velocity of the center of mass of PC molecules in liquid PC at* T* = 300 K. For this strongly bound liquid, the velocity acf relaxes through negative values after several collision times. Consistent with this, the friction kernel $\gamma \left( t\right)$ relaxes over many collision times. This behavior has been attributed to attractive intermolecular interactions in these strongly bound liquids. (*top-right*) Mean-squared displacements of PC in liquid PC (*dashed*). The time derivative (following Eq. 3, *blue solid curve*) shows a prominent maximum due to the negative tail of the velocity autocorrelation function. After that maximum, the msd achieves a constant slope only slowly. This behavior is not evident, for example, in the hard-sphere liquid model [46]. (*bottom*) Similar plots for EC

### Molecular Mobilities

**Fig. 6:**
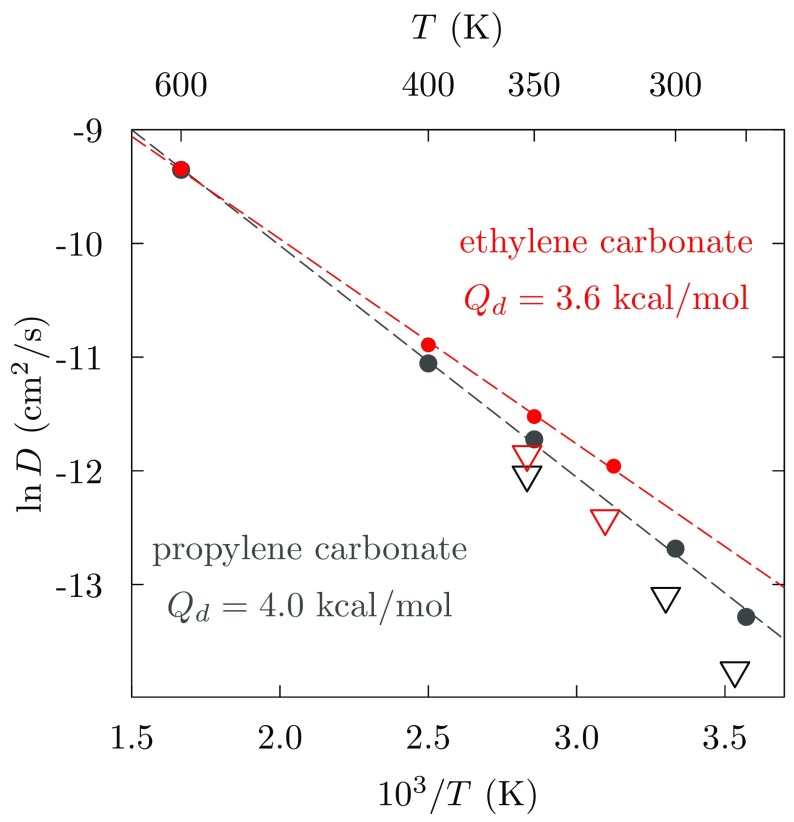
Comparison of simulation [48] and experimental [70] values of EC/PC molecule self-diffusion coefficients, over a range of temperatures at constant ambient pressure. The experimental results were extracted from studies of LiPF$_6$ solutions. $Q_\mathrm{d}$ is the activation energy parameter, ln $D \propto -Q_\mathrm{d}/k_{\mathrm {B}}T$, identifying the slope of the indicated fitting lines

Here we characterize the mobilities of EC and PC molecules in their liquid by the slope of the mean-squared displacement (msd)

1 $$\begin{aligned} \frac{\mathrm {d}\left\langle \Delta r\left( t\right) ^2\right\rangle }{\mathrm {d}t} \sim 6 D~, \end{aligned}$$

at long times *t*, thus evaluating the self-diffusion coefficient, *D*. But we can take that characterization deeper (Fig. 5) before considering those mobilities broadly (Fig. 6). The step deeper is to consider the velocity autocorrelation function (acf) [67]

2 $$\begin{aligned} C(t) = \left\langle \mathbf {v}\left( 0\right) \cdot \mathbf {v}\left( t\right) \right\rangle /\left\langle v^2\right\rangle ~, \end{aligned}$$

from which the mobilities

3 $$\begin{aligned} \frac{\mathrm {d}\left\langle \Delta r\left( t\right) ^2\right\rangle }{\mathrm {d}t} = 2\left\langle v^2\right\rangle \int _0^t C\left( \tau \right) \mathrm {d}\tau , \end{aligned}$$

may be then derived. Here the indicated velocities are those of the center of mass of the polyatomic EC/PC molecules. We also consider the friction kernel $\gamma (t)$ defined by [67]

4 $$\begin{aligned} m \frac{\mathrm {d}C(t)}{\mathrm {d}t} = -\int _0^t \gamma (t-\tau )C(\tau ) \mathrm {d}\tau ~, \end{aligned}$$

with *m* as the mass of the molecule. $\gamma (t)$ characterizes the random forces on these molecules, and

5 $$\begin{aligned} \gamma (0) = m\Omega ^2 = \left\langle F^2\right\rangle /3k_{\mathrm {B}}T~, \end{aligned}$$

emphasizes that connection with the forces on the molecules with $\Omega ^2 = \left\langle F^2\right\rangle /3mk_{\mathrm {B}}T.$ An interesting observation for these strongly bound liquids [46, 47, 68] (Fig. 5) is that $C\left( t\right)$ exhibits a negative tail, i.e., relaxation through negative values for times longer than a collision time, and that negative tail substantially affects the evaluation of *D* through Eq. (3). Contrary to the standard Langevin picture [67], the friction kernel $\gamma (t)$ also persists in relaxation over the same timescales of many collision times. That longer-timescale relaxation has been attributed to attractive intermolecular interactions in these strongly bound liquids [46, 47, 68, 69], particularly for the mobility of ions in solution for which long-ranged attractive forces are defining qualities. For the neutral PC molecule, indeed, that slowly relaxing tail of $\gamma \left( t \right)$ diminishes for the highest *T*s considered [46, 47].

Experimental results for *D* are only available for solutions of EC and PC with LiPF$_6$ at 1M concentration [70]. Nevertheless, here we compare our computed results [48] to those mobilities (Fig. 6). Our results agree with those experimental values to within about a factor of 2, satisfactory accuracy here. This encouraging comparison supports the use of the present non-polarizable force field in the studies reviewed below. The temperature dependence of $\ln D$ is linear in 1/*T* over the range considered.

**Fig. 7:**
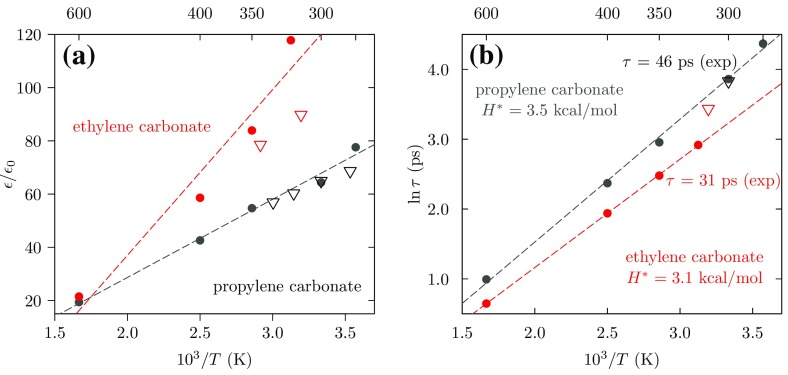
**a** Static dielectric constants $\epsilon /\epsilon _0$ (*in open triangles*) and **b** relaxation times $\tau$ for EC (*black*) and PC (*red*) at several temperatures. The experimental relaxation times indicated are those provided in the figure captions of the available experimental report [71], which incorporates an estimate of molecular polarizability and assumes a Debye relaxation model. *Dashed lines* are linear fits to the data, provided for visual guidance

### Dielectric Constants and Relaxation Times

The high dielectric constants of EC and PC liquids correlate naturally with the solubility of strong electrolytes, including lithium salts, in these solvents [72]. Dielectric characteristics are thus properties of first interest for these liquids. Dielectric constants and relaxation times are strongly temperature-dependent, and that might have consequences for battery efficiency and safety. Here, the computed static dielectric constants (Fig. 7a) are in good agreement with the experimental values for both PC and EC [44, 48, 62, 65], though at the lowest temperature here, the discrepancy is nearly 30% (too large) for EC.

Dielectric relaxation characterizes the ability of the material polarization to follow a changing applied electric field [73]. Harmonic analysis of the field and the polarization leads to a frequency-dependent, complex dielectric constant [73–75]

6 $$\begin{aligned} \epsilon (\omega )=\epsilon '(\omega )-i\epsilon ''(\omega ). \end{aligned}$$

with real and imaginary parts. $\epsilon {''}(\omega )$ describes frictional energy loss, and can be obtained from the polarization autocorrelation function [71, 75–77]

7 $$\begin{aligned} P(t) = \langle M(0)M(t)\rangle / \langle M^2\rangle , \end{aligned}$$

of the total dipole moment at time *t*, *M*(*t*), of the liquid. The acf *P*(*t*) is then fit to the Fourier transform of a stretched exponential (or Kohlrausch-Williams-Watts, KWW) model [78],

8 $$\begin{aligned} P_{\mathrm {KWW}}(t) = \exp [-(t/\tau )^\beta ], \end{aligned}$$

where $\beta$ is the fitting parameter. Available experimental data and analysis of simulation data suggest that $\beta = 1$ (Debye relaxation) is an accurate approximation for these systems.

The agreement between computed and experimental relaxation times is encouraging: $\tau$ = 46 ps from experiment on PC at room temperature compared to $\tau$ = 48 ps from these simulations. Note that extraction of the experiment relaxation times utilized models incorporating electronic polarizability $\epsilon (\omega = \infty )/\epsilon _0$, which we deliberately avoid here. Still, the relaxation times of 46 ps for PC, 31 ps for EC, and 8 ps for water emphasize [77] the comparative sluggishness of the carbonate solvents. This comparative sluggishness presents a severe challenge for simulation of these liquids on the basis of the more demanding simulation techniques such as AIMD.

The temperature dependence of the relaxation times

9 $$\begin{aligned} \tau ^{-1} = A~\exp (-H^*/{k_{\mathrm {B}}T}), \end{aligned}$$

can be modeled with an activation energy, $H^*$. For simplicity, we assumed the pre-exponent factor *A* to be independent of *T* [71, 79, 80], and calculate $H^*$ from the slope of the Arrhenius plot,

10 $$\begin{aligned} \ln \tau \propto H^*/k_{\mathrm {B}}T~. \end{aligned}$$

The computed $H^*$ for EC (3.1 kcal/mol) and PC (3.5 kcal/mol) are within the range of activation enthalpies reported for liquid water ($2.8-4.5$ kcal/mol for $278 \mathrm {K}< T < 348$ K) [81].

Though the present computational results cover a broad temperature range, fitting beyond a single activation energy has not been warranted so far. Still, it would be interesting, and maybe of practical relevance, for subsequent experiments and modeling to investigate super-cooled conditions more thoroughly.

**Fig. 8:**
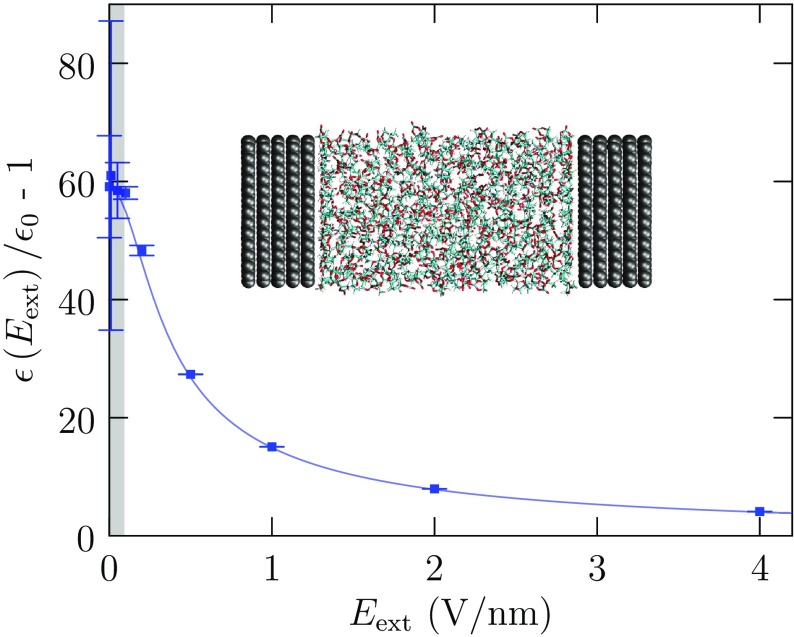
For uniform liquid PC, $\varepsilon \left( E_{\mathrm {ext}}\right) /\varepsilon _0 - 1$ as a function of external electric field strength, redrawn from Yang et al. [82]. These molecular dynamics results were obtained for the uniform liquid with an applied uniform electric field. The *error bars* indicate a 95% confidence interval. The curve is the fitted Booth model [62, 83–86]. The *shaded region* identifies the low-field regime based on the Booth model and that empirical parameterization. Evidently, the high-field behavior is simple in the model and the simulation. These calculations have been reexamined [87], and refined and extended [62]. Distinct from these uniform liquid calculations [82], the inset suggests how molecular-scale electric fields might be approached, i.e., by investigation of common lab-scale potential changes over nanometer-scale gaps

### Non-linear Polarization Response

The molecular electric fields at play on a molecular scale in ionic solutions are often much stronger than the laboratory electric fields used to measure dielectric constants. Thus, the equilibrium polarization responses to strong fields (Fig. 8) are often queried, even though statistical mechanical theories are less firmly grounded then. Indeed, the underlying theory of non-linear polarization response has been reexamined recently from a basic perspective [88–90]. Still, it is now clear that long-standing simple models [83] can do a good job of fitting non-linear polarization responses in controlled settings [62, 84, 91]. Interesting recent work [62] studied PC, EC, dimethyl carbonate (DMC), acetonitrile, and EC/DMC mixtures, and observed electrofreezing in several of these cases.

**Fig. 9:**
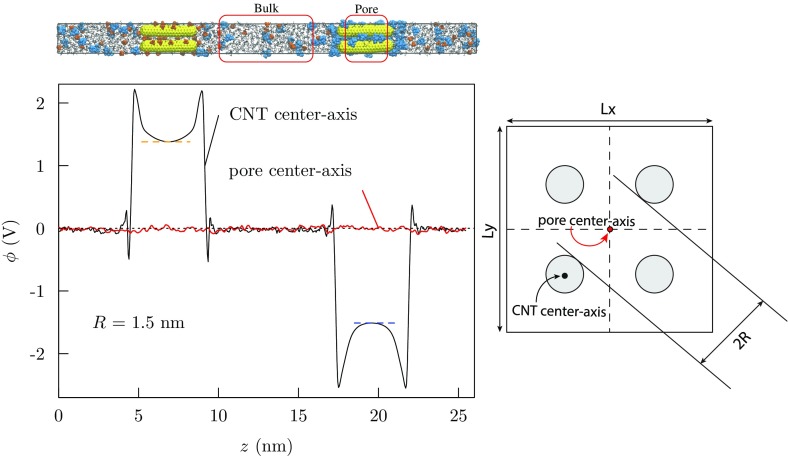
*Left top*: a snapshot of a simulation box [47, 92] containing 4 positively (*left*) and 4 negatively (*right*) charged CNTs, each of 360$^\circ$ C atoms, with ions filling the pore regions around CNT. A total charge of $q = \pm N$ e is set on each nanotube with *N* = (2, 4, 6, 8, 10) and a solution of 1 M TEABF$_4$ (tetraethylammonium tetrafluoroborate) electrolyte (*blue* and *orange*, respectively) in propylene carbonate (*sticks*). The highest charged case, then, has 223 C/gm, or adoption of 1300 m$^2$/gm as a standard value for the specific area, 0.17 C/m$^2$. The electrostatic potential was evaluated from the observed average charge density by numerical solution of the discretized Poisson equation [93]. *Left bottom*: a variation of electrostatic potential along the *z*-axis for a pore radius of *R* = 1.5 nm at a charge level of *N* = 6. *Right*: a cross-section perpendicular to the *z*-axis defining the pore radius and the location of pore and CNT axis

### Electrochemical Double-Layer Capacitor Based on CNT Forests

Beyond clear potential for practical significance, EDLCs based on CNT forests offer the possibilities of better molecular-scale understanding of those solutions in contact with charged electrodes. This possibility is enabled by the simplicity of the electrode chemistry and the fact that the microstructures of CNT forests can be simple and controlled over interesting ranges [94]. Thus EDLCs based on CNT forests provide a comparatively simple and controllable setting to learn about the molecular solutions. Outstanding practical questions that call for better basic molecular-scale understanding include (a) the dependence of the capacitance on electrostatic potential [92, 94] and (b) the dependence of capacitance on pore sizes for mesoporous electrode materials [95, 96]. Conclusive examination of those interesting questions will have to await further considerations. Here we make some primitive observations on work available so far.

One consideration for simulating these systems is the modeling of the electrodes. The simple model exemplified in Fig. 9 sets fixed charges based on appropriate preliminary calculations [47, 92]. An alternative focuses on the conducting nature of the electrode and reformulates the simulations to incorporate a constraint of constant electric potential in a conducting phase [97–102]. The work of Wang et al. [97] studying LiClO$_4$/acetonitrile between planar graphite electrodes with a constant potential MD calculation provided a clarifying example. The distribution of the fluctuating charges on electrode atoms was simple (unimodal) for cases exploring a voltage window below 4 V, though that situation changed markedly for net electric potential differences between the electrodes of 4 V and above. We note in passing that 4 V is close to the practical limit for the voltage window for experimental EDLC cases [94]. The complexities observed with the ultra-high potentials were associated with the depletion of the acetonitrile occupancy in Li$^+$ inner shells for Li$^+$ ions in close contact with the electrode. Those close contacts qualitatively change the density profiles of Li$^+$ ions with respect to the electrode, but do not qualitatively change the density profiles of the bigger ClO$_4{}^-$ ion.

The physical conclusion is that the EDLC/CNT calculations discussed above with molecular ions (Fig. 9) conservatively aim for a range in which they provide a reasonable initial step, perhaps subject to subsequent refinement. We note additionally that applications of constant potential MD calculations, implemented in simulation packages such as LAMMPS [103], have been limited so far in the number of electrodes and their configurations, specifically to two-planar electrodes. We take up the important special case of Li$^+$ in the next section.

**Fig. 10:**
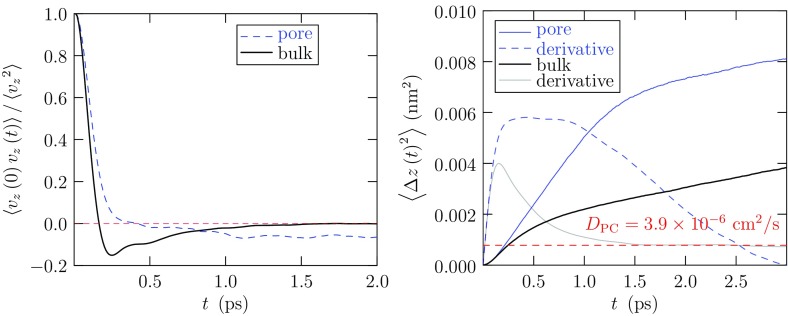
Comparison of mobilities of PC molecules in bulk solution with PC molecules in the pore space of the CNT forest (Fig. 9) [47]. In this case, the CNTs are *not* charged and the pore radius (Fig. 9) is *R* = 1 nm. The indicated value of *D* (*right panel*) is for PC molecules in the bulk solution. Notice that the mobility of PC molecules in the pore looks diffusive at intermediate times, but not for longer times here. Further, the suggested intermediate-time diffusive motion is faster in the pore, presumably an effect of preferential orientation of PC molecules in the pore space

We emphasize that realistic molecular models of EDLC/CNTs are feasible for direct numerical simulation of the pore filling and the electrical characteristics [47, 91, 92]. This reduces possible uncertainty about solution composition in the pore spaces and permits study of the kinetics of the filling in realistic settings. That the relevant molecular mobilities are different in the pore spaces is already clear (Fig. 10).

## Empirically Scaled Partial Charges for Li$^+$...Carbonate Interactions

The results discussed in previous sections suggest that a parameterized, non-polarizable force field can reproduce experimental structural, thermodynamic, and dielectric properties of EC and PC liquids with acceptable accuracy [44, 46–48]. Next we consider the important case of addition of Li$^+$ ions to EC and PC. A primary concern is a valid description of the thermodynamics of Li$^+$-solvent interactions. In view of the strength of those interactions, and non-linear behaviors exhibited in Fig. 8, these thermodynamic issues are not taken for granted. In setting revised models, we considered partial charges, empirically scaled on the basis of electronic structure calculations and available experimental results. The electronic structure-based methods employed [50] are (a) quasi-chemical theory for the thermodynamics, and (b) AIMD calculations for structural and mobility information.

We have compared results using partial charges available in the standard OPLS-AA distribution for EC and PC solvents [104] to results derived from partial charges that were subsequently reduced to 90 and 80% of those values. Simulations treated a single Li$^+$ ...PF$_6{}^-$ ion pair in both solvents. We compared structural and thermodynamics results with chemically based AIMD simulations.

### Free Energy Results and Quasi-Chemical Theory (QCT)

QCT is based on the study of the occupancy of an inner shell of an Li$^+$ ion, here, by the carbonyl O atoms of the solvent. QCT provides the free energy, specifically the excess chemical potential, $\mu ^{\mathrm {(ex)}}_{\mathrm {Li}^+}$, for a solution phase Li$^+$ [50]. We use the cluster QCT method [105, 106]

11 $$\begin{aligned} \mu ^{\mathrm {(ex)}}_{\mathrm {Li}^+} = -kT\ln K^{(0)}_{n}\rho _{\mathrm {sol}}{}^n + \left( \mu ^{\mathrm {(ex)}}_{\mathrm {Li(sol)}_n{}^+}-n\mu ^{\mathrm {(ex)}}_{\mathrm {sol}}\right) , \end{aligned}$$

as a benchmark for comparison of QCT results for $\mu ^{\mathrm {(ex)}}_{\mathrm {Li}^+}$ obtained from MD simulation with simple force fields. In the first term, $K^{(0)}_n$ is the equilibrium ratio for the Li$^+$-solvent (sol) association reaction

12 $$\begin{aligned} \mathrm {Li}^{+} + n~\mathrm {sol} \rightleftharpoons \mathrm {Li(sol)}_n{}^{+}, \end{aligned}$$

treated as in an ideal gas phase; hence the superscript (0). The solvent density, $\rho _{\mathrm {sol}}$, gauges the availability of solvent molecules to serve as ligands in this association, and this justifies the attention above to the equation of state of these liquids. The right-most term of Eq. (11) provides solvation of the $\mathrm {Li(sol)}_n{}^{+}$ complex by the solvation environment external to it. That, $\mu ^{\mathrm {(ex)}}_{{{\rm Li}({\rm sol})_{n}{}^{+}}}-n\mu ^{{({\rm ex})}}_{{\rm sol}},$ combination makes a favorable contribution to the free energy.

For analyzing the MD results, we use the direct QCT approach [105]

13 $$\begin{aligned} \mu ^{\mathrm {(ex)}}_{\mathrm {Li}^+}/RT = -\ln p^{(0)}(n_{\lambda }) + \ln \left\langle {e}^{\varepsilon /RT}\mid {n_{\lambda }}\right\rangle + \ln p(n_\lambda )~, \end{aligned}$$

which is tautologically related to Eq. (11) with the natural definition of the indicated probabilities [107]. $\varepsilon$ is the binding energy of the Li$^+$. The advantage of this simulation-based QCT is that it permits calculation of solvation free energies, and correlation of those results with observed solution features.

**Fig. 11:**
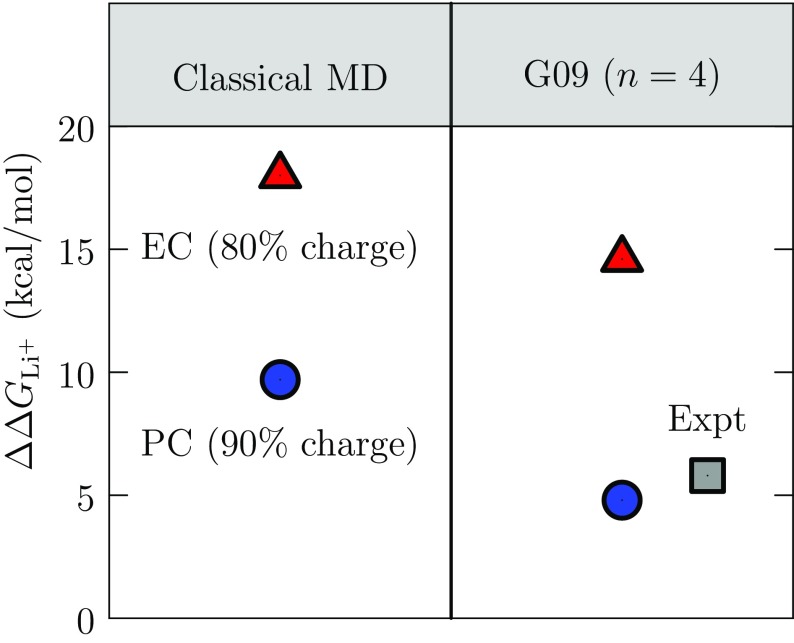
Transfer free energies, $\Delta \Delta G_{\mathrm {Li}^+}$, comparing FFMD direct QCT results (*left*) and cluster-QCT results (*right*) using the G09 electronic structure software package. The cluster QCT results for the free energy of Li$^+$ transfer to PC from water agree with tabulated experimental values to within 1 kcal/mol [108]. Our experience using cluster QCT to predict ion hydration free energies [109–119] suggests that the G09 results have accuracy comparable to other $ab \, initio$ predictions [120–123], and hence provide a useful benchmark for FFMD results

The free energies of Li$^+$ transfer $\Delta \Delta {G_{\mathrm {Li}^+}}$ to a carbonate solvent from water for the two QCT implementations (Fig. 11) compare accurate electronic structure calculations and classical FFMD simulation with simple force fields. The cluster QCT result for the free energy of Li$^+$ transfer to PC from water agrees with the value tabulated by Marcus [108]. No experimental value is available for EC. The direct QCT evaluations of $\Delta \Delta {G_{\mathrm {Li}^+}}$ for the FFMD simulations agree reasonably with the cluster QCT electronic structure calculations when the partial charges of the force fields are scaled by 80% (EC) or 90% (PC). The direct QCT MD calculations agree to within 2 kcal/mol on the 10-kcal/mol difference in transfer free energies between PC and EC predicted by the cluster QCT calculations.

Positive transfer free energies favor lithium ion solvation by water compared with either carbonate solvent. From the perspective of the cluster QCT calculations, the replacement free energy, reflecting the availability of the solvent molecules as ligands, is the foremost factor leading to that result. Comparing EC and PC transfer free energies, again from the perspective of cluster QCT, the solvation of the bare EC/PC molecules serving as ligands is decisive in arriving at a positive free energy of transfer from PC to EC, with EC being slightly smaller.

**Fig. 12:**
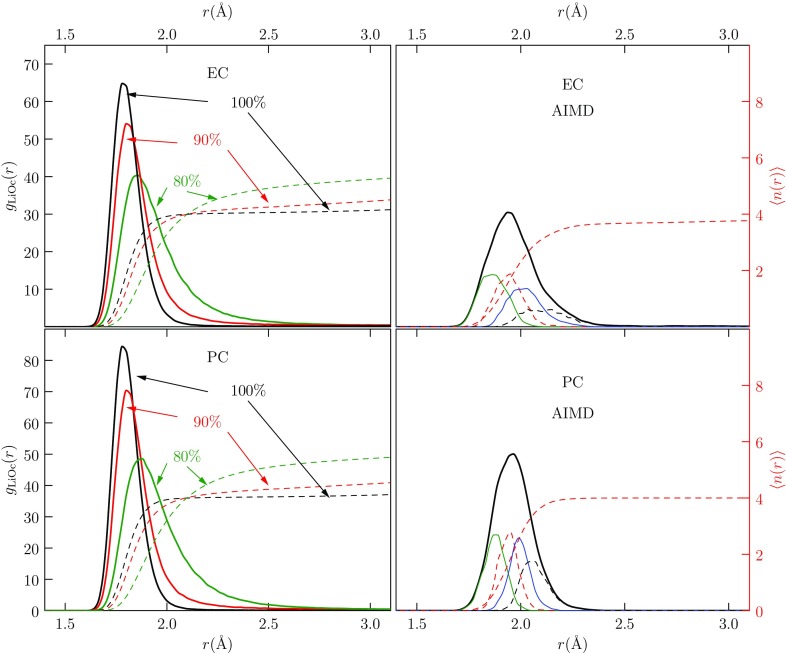
Radial distributions of carbonyl O atoms from Li$^+$ in (*top*) EC and (*bottom*) PC using (*left*) FFMD and (*right*) AIMD simulations. Running coordination numbers (*dashed curves* and *right axes*) show near-neighbor (inner-shell) occupancies. In the FFMD cases, partial charges on EC and PC molecules were reduced from 100 to 90%, and subsequently to 80%. AIMD results show that four solvent molecules fully saturate the Li$^+$ coordination. The FFMD results demonstrate the importance of repulsions between near-neighbor (inner-shell) solvating molecules: the occupancy of the inner shell increases moderately, and inner-shell structures broaden, as the solvent partial charges are scaled down

### Radial Distribution Function

Radial distributions (rdfs) of carbonyl O of EC and PC with Li$^+$ are sensitive to partial charges of an FFMD model (Fig. 12). AIMD results of Li$^+$ solvation in water [50] and PC agree with x-ray spectroscopy [34] and neutron diffraction [124, 125] results. Hence, we used AIMD for validation of FFMD results. The four-coordinate inner shell was observed in both AIMD and FFMD simulations of Li$^+$ solvation in EC and PC. The AIMD results match those from previous calculations [126]. The coordination number of Li$^+$ in PC using AIMD agrees with the 4.5 reported by neutron diffraction [125] and x-ray spectroscopy [34]. Interestingly, Bader charge analysis on AIMD configurations suggest that solvent molecules sometimes donate as much as 0.1 electron to an ion [29, 127]. The neutron and x-ray diffraction experiments have the peak position at 2.04 ${\AA }$, which is slightly longer than all FFMD results (1.78–1.9 ${\AA }$), but comparable to AIMD (2 ${\AA }$) and polarizable force field results (1.95–2 ${\AA }$). Yet, a four-coordinate inner solvation shell is seen consistently in all cases. The solvent density is less sensitive to these partial charges under the conditions of interest. Dielectric constants do change significantly with scaled partial charges, but the values realized are high enough that solvation characteristics are only slightly affected.

### Ion Mobilities

The Li$^+$ msd results [128] were obtained from separate 1-ns simulations of one Li$^+$...PF$_6^-$ ion pair in 249 EC and PC molecules, and compared with the experimental values obtained from nuclear magnetic resonance (NMR) results [70]. The results (Fig. 14) for 90% charged PC and 80% charged EC were closest to the experimental results. The transference number, $t_{\text {Li}^+}$, for Li$^+$ can be calculated from ratios of diffusion constants, *D*, according to

14 $$\begin{aligned} t_{\text {Li}^+} = \frac{D_{\mathrm {Li}^+}}{D_{\mathrm {Li}^+}+D_{\mathrm {PF}_6{}^-}} . \end{aligned}$$

**Fig. 13:**
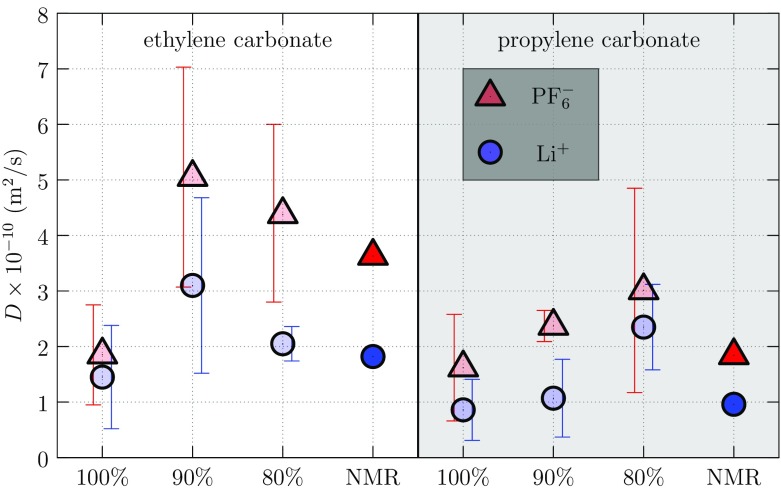
Diffusion constants were calculated from FFMD using the mean-squared displacement (msd) of Li$^+$ and PF$_6{}^-$ ions in EC (*left*) and PC (*right*). The msd was calculated for 25 ps. Experimental values are taken from Hayamizu et al. [70] As evident from these plots, the diffusion constants match experimental values best for 80% of partial charges on EC and 90% on PC

The computed transference numbers of 0.35 (EC) and 0.31 (PC) are consistent with previous NMR [70] and impedance spectroscopy (EIS) [128] experiments. The diffusion constant value changes significantly with partial charges of solvent but the transference numbers are less sensitive.

**Fig. 14:**
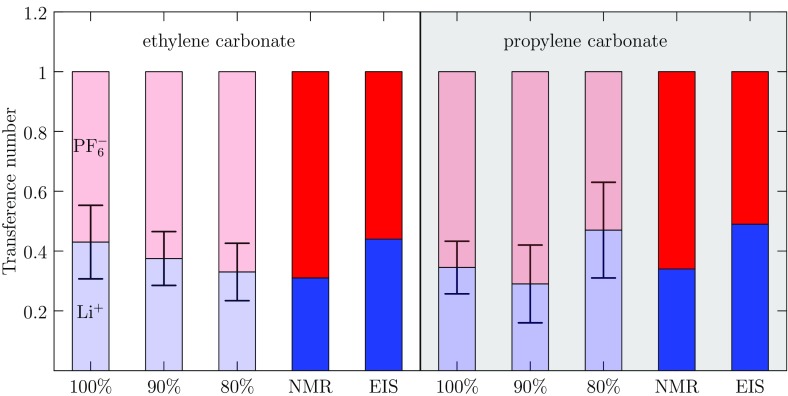
Transference numbers for Li$^+$ and PF$_6^-$ in EC (*left*) and PC (*right*) were calculated using FFMD and Eq. 14. The NMR diffusion constants at 1 M salt concentration were used to calculate experimental transference numbers (NMR) [70] and the combined AC impedance and DC polarization results apply to 0.1 M salt concentration (EIS). The experimental results show little dependence on salt concentration, and the calculated results agree with the experimental numbers

In summary, changes to the partial charges on PC and EC solvents alter solvation structure and transport properties of Li$^+$ and PF$_6^-$ ions. Based on our results for radial distribution functions (Fig. 12), diffusion constants (Fig. 13), and transference numbers (Fig. 14), we identify 90% scaling of PC partial charges and 80% scaling for EC.

**Fig. 15:**
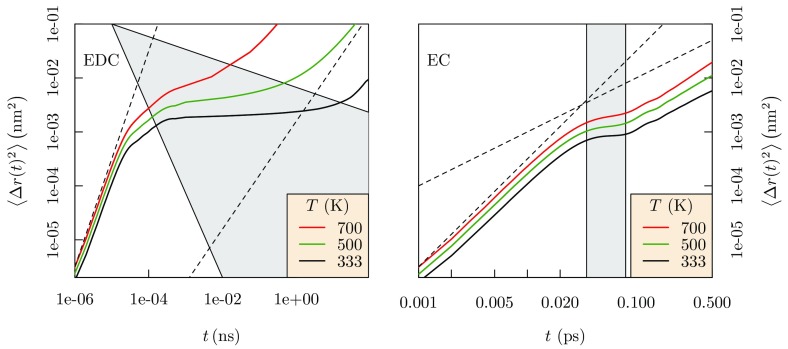
Mean-squared displacements for Li$^+$ in EDC and EC. The behavior in EDC at intermediate timescales $0.001~\mathrm {ns}< t < 1~\mathrm {ns}$ demonstrates trapping of the Li$^+$ ion (*gray shaded area*). Ballistic motion is evident at short timescales, and diffusive motion at long timescales in both EDC and EC solvents. At high *T*, the trapping regime shrinks. Note the time scales differ dramatically between the left and right panels here. In the model SEI (*left panel*), trapping times are longer than nanoseconds. Thus for direct simulation of trapping, simulations should access multiples of that nanosecond time scale

## Model solid electrolyte interphase layer

The discussion above encourages us to apply such an empirical non-polarizable force field to study non-aqueous electrolytes more broadly. Thus we extended our effort to simulate Li$^+$ ion transport within a model SEI layer of dilithium ethylene dicarbonate (EDC) [129]. The Li$^+$ ion msd in EDC and EC solvents (Fig. 15) shows three distinct temporal regions corresponding to ballistic, trapping, and diffusive motions. The trapping region for Li$^+$ ion is extended for glassy EDC material and has significant temperature dependence compared to liquid EC solvent. Further analysis [129] confirmed the glassy behavior of the EDC matrix.

## Conclusions

The basic results discussed suggest that an empirically parameterized, non-polarizable force field can reproduce experimental structural, thermodynamic, and dielectric properties of EC and PC liquids with acceptable accuracy. More sophisticated force fields might include molecular polarizability and Buckingham-model description of inter-atomic overlap repulsions as extensions of Lennard-Jones models of van der Waals interactions. Simple approaches should be similarly successful also for applications to organic molecular ions in EC/PC solutions, but the important case of Li$^+$ deserves special attention because of the particularly strong interactions of that small ion with neighboring solvent molecules. To treat the Li$^+$ ions in liquid EC/PC solutions, we identify interaction models defined by empirically scaled partial charges for ion-solvent interactions. The empirical adjustments use more basic inputs, electronic structure calculations and AIMD simulations, and also experimental results on Li$^+$ thermodynamics and transport in EC/PC solutions. Application of such models to the mechanism of Li$^+$ transport in glassy SEI models emphasizes the advantage of long time-scale molecular dynamics studies of these non-equilibrium materials.
